# *Fusarium* diversity from the Golden Gate Highlands National Park

**DOI:** 10.3389/fmicb.2023.1149853

**Published:** 2023-04-13

**Authors:** Carla Steyn, Adriaana Jacobs, Brett Summerell, Eduard Venter

**Affiliations:** ^1^Department of Botany and Plant Biotechnology, University of Johannesburg, Johannesburg, South Africa; ^2^ARC-Plant Health and Protection, Agricultural Research Council, Pretoria, South Africa; ^3^Australian Institute of Botanical Science, Royal Botanic Gardens and Domain Trust, Sydney, NSW, Australia

**Keywords:** *Fusarium* diversity, Afromontane, grassland biome, natural ecosystems, species complex, low anthropogenic disturbance

## Abstract

Members from the genus *Fusarium* can infect a broad range of plants and threaten agricultural and horticultural production. Studies on the diversity of *Fusarium* occurring in natural ecosystems have received less attention than the better known phytopathogenic members of the genus. This study identified *Fusarium* species from soils with low anthropogenic disturbance found in the Golden Gate Highlands National Park (GGHNP), a part of the Drakensberg system in South Africa. Selective techniques were implemented to obtain 257 individual isolates from the selected soil samples for which the *translation elongation factor 1*α (*tef-1*α) gene region was sequenced and compared against the *Fusarium* MLST and *FUSARIUM*-ID databases. Phylogenetic analyses, based on maximum likelihood and Bayesian inference, were used to determine species diversity in relation to reference isolates. Species level identifications were made within three of the seven species complexes and identified *F. brachygibbosum*, *F. sporotrichioides*, *F. andiyazi*, and *F. gaditjirri* based on the *FUSARIUM*-ID database, with *F. transvaalense* and *F. lyarnte* identified against the *Fusarium* MLST database. This indicated highly diverse populations of *Fusarium* from soils with low anthropogenic disturbance from the Afromontane grassland region found in mountain ranges.

## Introduction

The genus *Fusarium* evolved from the ancient lineage of ascomycetous organisms (Taylor and Berbee, 2006) and includes a significant variety of morphological and phylogenetic characters that is indicative of early differentiation. The phytopathogenic *Fusarium* species may have co-evolved with their associated plant hosts but this is not always applicable (Wang et al., 2004; Summerell et al., 2010). Taylor and Berbee (2006) developed a dating system for the divergence of important classes in the fungal kingdom, which included the genus *Fusarium*. These approximate divergence dates varies when different taxonomic groups are used as calibration points. The predicted divergence of the genus *Fusarium* is estimated between 110 and 250 to 420 million years ago (Taylor and Berbee, 2006). Based on two of the three calibration models tested by Taylor and Berbee (2006), the genus *Fusarium* diverged well before the splitting of the Pangaea supercontinent. This supports the early distribution and diversity of *Fusarium* members found in varying climates across the world. Thus, host association coupled with climatic regions may be considered as influencing factors that indicate the origin of *Fusarium* species recovered from specific geographic locations (Summerell et al., 2010). Although with the wide and cosmopolitan distribution of the genus *Fusarium*, it is still difficult to establish the initial point of origin (Summerell et al., 2010).

As *Fusarium* species form part of ecosystems all around the world, their distribution and the link to biogeographic development provides an opportunity to acquire information on speciation and dispersal effectors (Summerell et al., 2010). The main focus of phylogeography is to determine species distribution and possible mechanisms that result in speciation events (Hickerson et al., 2010). Phylogeographic studies are widely used in conservation and may also be used to identify cryptic lineages that are not always clearly indicated by morphological variation (Wiens, 2012). Fungal (microbial) and animal biodiversity is greatly influenced by the number and community relationship of plant species available in the area that is in turn influenced by soil features such as the pH and climatic factors, particularly the availability of water, especially in an area with varying altitudes (Shen et al., 2013).

South Africa possesses nine different biomes (Mucina and Rutherford, 2006). The grassland biome is critically important for food production as it is used for commercial agriculture, feeding grounds for livestock, or for subsistence farming (Rademeyer and van Zyl, 2014). It has been reported that 33% of the temperate grasslands are already irreversibly transformed (Carbutt et al., 2011) and that only 36.7% is under conservation (Reyers et al., 2005). Within the Golden Gate Highlands National Park (GGHNP) the two bioregion types, Mesic Highveld Grassland Bioregion and The Drakensberg Grassland Bioregion are found (Rutherford et al., 2006). The Mesic Highveld Grassland Bioregion is the largest of the four grassland bioregions and contains a highly diverse vegetation community that includes bushveld and summit grasslands. In contrast to the Mesic Highveld Bioregion, the Drakensberg Grassland Bioregion has the lowest number of vegetation types. The Drakensberg Grassland Bioregion stretches southward from the Lesotho highlands along the high-lying escarpment area in the Eastern Cape Province, it occurs at a higher altitude than comparative bioregions with a greater occurrence of frost (Rutherford et al., 2006). The Drakensberg Grassland Bioregion is most conserved due to efforts by the Maloti-Drakensberg Transfrontier Conservation Area and the Mesic Highveld Grassland Bioregion has been transformed by 42.91% with its conservation status indicated as “very urgent” (Carbutt et al., 2011). The GGHNP is an important site for tourism after its establishment in 1963. The region was used as farmland before being proclaimed as a national park. The soil types of the park are highly fertile, especially in regions lying above 2 070 m, and supports dense temperate grassland that in turn supports the steep slopes and minimises the effects of erosion. In some areas, the shallow sandy soil are more prone to erosion (Roberts, 1969). For the current study it was hypothesised that the *Fusarium* diversity found in this area/habitat (grassland with low anthropogenic disturbance) would be limited and that the species distribution would be different to that observed in the lower lying grassland biome. The possibility of recovering new previously undefined species would also be likely.

Three new species of *Fusarium* were identified in the Kruger National Park, South Africa, by Sandoval-Denis et al. (2018). The species were described as *F. fredkrugeri* (Sandoval-Denis et al., 2018), *F. convolutans* (Sandoval-Denis et al., 2018) and *F. transvaalense* (Sandoval-Denis et al., 2018) and were isolated from the rhizosphere of native herbaceous plant species endemic to the area (Sandoval-Denis et al., 2018). They were isolated from points with differing altitudes and soil characteristics across a catena slope and is an example of the variation in biogeography on the distribution of species. An earlier, study by Burgess and Summerell (1992), looked at the geographical distribution in Queensland, Australia, of *Fusarium* species obtained from subtropical and semi-arid grassland soils. The majority of the isolates comprised of *F. chlamydosporum*, *F. compactum* and *F. equiseti*, with *F. chlamydosporum* and *F. equiseti* recovered mostly from the drier sampling areas, although it is worth noting that identification was based on morphological features and cryptic species may not have been recognised. Chehri et al. (2010) found that *F. chlamydosporum* is associated with soils from warmer regions, and *F. sporotrichioides* and *F. sambucinum* is associated with soils from colder regions.

It is important to be able to fully grasp the biodiversity in a specific environment, especially one as important as the grassland biome, which supports many livelihoods. The focus of the current study was to ascertain the natural threshold of *Fusarium* species found in the semi-disturbed soils of the Afromontane grassland region of the GGHNP. This will contribute to ongoing research on the distribution of fusaria from the South African grassland biome (Jacobs-Venter et al., 2018; Mavhunga, 2021). The identification of soil borne species from this major phytopathogenic genus will expand our knowledge regarding possible origin of *Fusarium* species and their phylogeography.

## Materials and methods

### Soil sampling and culture isolation

**FIGURE 1:**
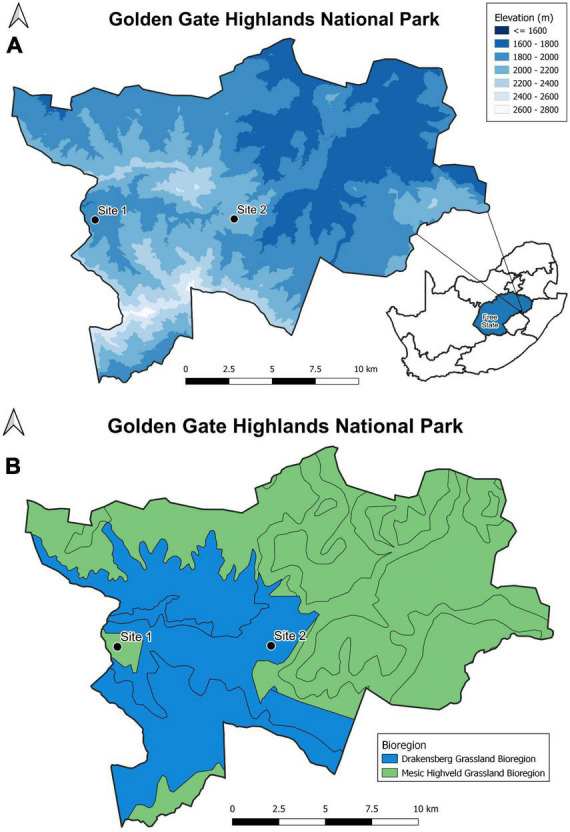
Map of the GGHNP, Free State Province, South Africa. **(A)** Elevation map indicating the collection sites used in this study. **(B)** Map of the Drakensberg and Mesic Highveld Grassland Bioregion types found at the GGHNP. Site 1 is in the Mesic Highveld and site 2 in the Drakensberg Grassland Bioregions.

The soil samples were collected from the Golden Gate Highlands National Park (GGHNP) that is located within the north-eastern part of the Free State province, South Africa. The grassland biome of the GGHNP is represented by both the Drakensberg Grassland Bioregion and the Mesic Highland Grassland Bioregion (Mabunda, 2011). The sampled sites, site 1 (28°30’41” S, 28°34’24” E; 1874 m; Mesic Highveld) and site 2 (28°30’39” S, 28°39’20” E; 2082 m; Drakensberg Grasslands) were selected for comparison based on their bioregion and elevation differences (208 m higher in elevation than site 1) (Figure 1). The sampling was done in the top 10 cm of the upper soil profile as this depth hosts the majority of soil micro-fungi (Mandeel, 2006) using a 15 m × 15 m standardised transect method (Laurence et al., 2012) and core sampler resulting in 5 samples pooled together to form a collective single sample for each site (Jacobs et al., 2018; Nephalela-Mavhunga et al., 2021). The collected soil was air dried at room temperature (20–25°C) overnight. From this larger collective sample, 5 g soil for each site was separated into large and small soil fragments using a 450 μm pore size sieve, resulting in the smaller fragments being ≤450 μm and the larger fragments ≥450 μm. Each soil fragment subsample, of the total separated 5 g, was evenly spread onto Spezieller Nährstoffarmer Agar (SNA) (Nirenberg, 1981; Leslie and Summerell, 2006) and incubated for 7 days at 25°C under near UV (black) light at a 12-h photoperiod to stimulate sporulation. Five fungal colonies displaying *Fusarium* morphology were picked from each plate and then sub-cultured to obtain pure individual isolates using single conidium transfer from 2% water agar onto quarter strength potato dextrose agar (PDA) (Nelson et al., 1983; Leslie and Summerell, 2006). This resulted in 257 isolates from which DNA was extracted after growth on full strength PDA (Lab M Limited) plates that were incubated for 5 days at 25°C under a 12-h near UV (black) light cycle. The isolates were assigned PPRI numbers (Jacobs et al., 2018) when deposited into the National Collection of Fungi (NCF), South Africa.

### DNA extraction, PCR and identification

DNA extraction for the individual isolates were executed using the *Quick*-DNA*™* Fungal/Bacterial Miniprep Kit (Zymo Research) according to the manufacturer’s protocol specifications. The DNA quality was evaluated using agarose gel electrophoresis before use as template in a PCR that amplified the *tef-1*α gene region comprising *EF*1 forward primer; 5’-ATGGGTAAGGA(A/G)GACAAGAC-3’ (Inqaba Biotechnical Industries (Pty) Ltd), 5 μM of *EF*2 reverse primer; 5’-GGA(G/A)GTACCAGT(G/C)ATCATGTT-3’ (Inqaba Biotechnical Industries (Pty) Ltd) (O’Donnell et al., 1998b). The *tef-1*α amplicons were sequenced for each of the 257 isolates. Sanger sequence reactions were manually edited and aligned using the MAFFT plugin incorporated in Geneious software (version 8.1.9) to produce a consensus sequence. Polymorphisms among the datasets were confirmed by examination of the electropherograms and gaps were regarded as missing data. The nBLAST™ analyses of the individual consensus sequences against the *FUSARIUM*-ID^1^ database (Geiser et al., 2004) and the *Fusarium* MLST^2^ database (O’Donnell et al., 2010) served as the first analysis to identify the relation of the GGHNP isolates to the different species complexes (O’Donnell et al., 2013). These identifications were recorded in Tables 1–7.

**TABLE 1:** The phylogenetic analyses for the identified species complexes and their substitution model and reference isolates sources tabulated.

Phylogenetic analysis	Isolation frequency	Substitution model	Reference isolates obtained from
FOSC	77%	GTR + G	O’Donnell et al., 1998b; O’Donnell et al., 2004, 2009; Laurence et al., 2014; Lombard et al., 2019a
FSAMSC	8%	GTR + G + I	Laraba et al., 2021
FIESC	7%	GTR + G + I	Xia et al., 2019
FFSC	4%	GTR + G	Yilmaz et al., 2021
Minor species complex	≤4%	GTR + G + I	O’Donnell et al., 2008, 2009, 2018; Laurence et al., 2011; Laurence et al., 2016; Sandoval-Denis et al., 2018

**TABLE 2:** Nucleotide BLAST (nBLAST™) results from the *Fusarium* MLST and *FUSARIUM*-ID databases for the FOSC.

PPRI no.	*Fusarium* MLST	*FUSARIUM*-ID	MYCOBANK similarity%	*FUSARIUM*-ID similarity*%*	Isolation site	Particle size	Accession number
24779	*F. oxysporum* f.sp. *melonis*	FOSC (222)	100%	100%	Site 1	Large	OL782448
24782	*F. oxysporum*	FOSC (231)	100%	99%	Site 1	Small	OL782557
24785	FOSC (18)	FOSC (18)	99%	99%	Site 1	Small	OL782526
24787	FOSC (239)	*F. oxysporum*	100%	100%	Site 1	Small	OL782525
25046	FOSC	FOSC (19)	100%	100%	Site 1	Small	OL782367
25047	FOSC	FOSC (19)	100%	100%	Site 1	Small	OL782372
25480	FOSC (18)	FOSC (18)	99%	99%	Site 1	Large	OL782502
25485	**FOSC (167)**	**FOSC (231)**	**99%**	**99%**	Site 1	Small	OL782558
25489	FOSC (222)	FOSC (222)	100%	100%	Site 1	Small	OL782447
25493	**FOSC (239)**	**FOSC (94)**	**99%**	**99%**	Site 1	Small	OL782503
25496	*F. oxysporum* f. *cubense*	FOSC (191)	100%	100%	Site 1	Small	OL782378
25497	FOSC	FOSC (191)	100%	100%	Site 1	Small	OL782454
25499	FOSC	FOSC (190	100%	100%	Site 1	Large	OL782471
25507	FOSC	FOSC (19)	99%	99%	Site 1	Small	OL782365
26238	*F. oxysporum*	FOSC (47)	100%	100%	Site 2	Large	OL782386
26247	*F. oxysporum* f. sp. *medicaginis*	FOSC (18)	99%	99%	Site 2	Small	OL782533
26265	*F. oxysporum* f. sp. *medicaginis*	FOSC (18)	99%	99%	Site 2	Large	OL782540
26268	*F. oxysporum*	FOSC (47)	99%	100%	Site 2	Large	OL782390
26269	FOSC (20)	FOSC (20)	100%	100%	Site 2	Small	OL782384
26274	*F. oxysporum* f. *cubense*	FOSC (191)	100%	100%	Site 2	Small	OL782381
26291	FOSC (18)	FOSC (18)	99%	99%	Site 2	Small	OL782537
26304	*F. oxysporum*	FOSC (222)	100%	100%	Site 2	Small	OL782441
26312	FOSC (47)	FOSC (47)	100%	100%	Site 2	Small	OL782435
26316	FOSC (179)	FOSC (179)	99%	99%	Site 2	Small	OL782554
26324	*F. oxysporum* f. sp. *medicaginis*	FOSC (18)	99%	99%	Site 2	Small	OL782539
26325	*F. oxysporum*	FOSC (191)	100%	100%	Site 2	Small	OL782371
26328	*F. oxysporum*	FOSC (18)	99%	99%	Site 2	Large	OL782518
26783	FOSC (18)	FOSC (18)	100%	100%	Site 2	Small	OL782493
26793	**FOSC (47)**	**FOSC (18)**	**100%**	**99%**	Site 2	Small	OL782388
27110	*F. oxysporum*	FOSC (18)	99%	99%	Site 2	Large	OL782481
27119	*F. oxysporum*	FOSC (47)	100%	100%	Site 2	Large	OL782389
27125	*F. oxysporum*	FOSC (18)	100%	100%	Site 2	Large	OL782512
27127	*F. oxysporum*	FOSC (18)	99%	99%	Site 2	Small	OL782482
27134	FOSC (191)	FOSC (191)	100%	100%	Site 2	Small	OL782427
27135	FOSC (191)	FOSC (191)	100%	100%	Site 2	Small	OL782428
27149	*F. oxysporum*	FOSC (18)	100%	100%	Site 2	Small	OL782514
27151	FOSC (191)	FOSC (191)	100%	100%	Site 2	Small	OL782429
27405	FOSC	FOSC (19)	99%	99%	Site 1	Large	OL782399
27422	*F. oxysporum*	FOSC (47)	100%	100%	site 1	Large	OL782436
27428	FOSC	FOSC (19)	100%	100%	Site 1	Large	OL782469
27429	*F. oxysporum* f. sp. *pisi*	FOSC (191)	99%	99%	Site 1	Large	OL782430
27430	**FOSC (239)**	**FOSC (94)**	**100%**	**100%**	Site 1	Large	OL782477
27431	**FOSC (167)**	**FOSC (231)**	**99%**	**99%**	Site 1	Large	OL782556
27496	FOSC (191)	FOSC (191)	100%	100%	Site 1	Large	OL782392
27502	*F. oxysporum* f.sp. *pini*	FOSC (91)	99%	99%	Site 1	Large	OL782439
27511	**FOSC (107)**	**FOSC (19)**	**99%**	**99%**	Site 1	Large	OL782402
27515	*F. oxysporum* f. sp. *pisi*	FOSC (191)	100%	100%	Site 1	Large	OL782419
27518	**FOSC (101)**	**FOSC (191)**	**100%**	**100%**	Site 1	Large	OL782462
27531	**FOSC (101)**	**FOSC (191)**	**100%**	**100%**	Site 1	Large	OL782460
27532	*F. oxysporum* f.sp. *pini*	FOSC (91)	99%	99%	Site 1	Large	OL782440
27538	**FOSC (101)**	**FOSC (191)**	**100%**	**100%**	Site 1	Large	OL782463
27542	FOSC (29)	FOSC (29)	100%	100%	Site 1	Small	OL782432
27543	*F. oxysporum* f.sp. *pini*	FOSC (91)	99%	99%	Site 1	Small	OL782438

Marked in bold are the isolates that produced contrasting nBLAST™ results between the two databases. Marked in gray are the isolates with identical MLST/haplotype types.

**TABLE 3:** Nucleotide BLAST (nBLAST) results from the *Fusarium* MLST and *FUSARIUM*-ID databases for the FSAMSC.

PPRI no.	*Fusarium* MLST	*FUSARIUM*-ID	*Fusarium* MLST similarity%	*FUSARIUM*-ID similarity%	Isolation site	Particle size	Accession number
26243	FSAMSC	*F. sporotrichioides*	94%	91%	Site 2	Small	OL782346
26246	FSAMSC	*F. brachygibbosum*	93%	93%	Site 2	Small	OL782338
26275	***Fusarium* sp.**	***F. sporotrichioides***	94%	91%	Site 2	Small	OL782342
26278	***F. transvaalense***	***F. brachygibbosum***	94%	93%	Site 2	Large	OL782335
26284	FSAMSC	*F. brachygibbosum*	94%	93%	Site 2	Small	OL782352
26285	FSAMSC	*F. sporotrichioides*	93%	91%	Site 2	Small	OL782353
26310	FSAMSC	*F. sporotrichioides*	94%	91%	Site 2	Small	OL782347
26311	FSAMSC	*F. brachygibbosum*	93%	93%	Site 2	Small	OL782340
26319	***Fusarium* sp.**	***F. sporotrichioides***	94%	91%	Site 2	Small	OL782344
26330	***F. transvaalense***	***F. brachygibbosum***	94%	93%	Site 2	Small	OL782348
26340	FSAMSC	*F. sporotrichioides*	94%	91%	Site 2	Small	OL782349
26771	***F. transvaalense***	***F. brachygibbosum***	94%	93%	Site 2	Large	OL782334
26782	FSAMSC	*F. sporotrichioides*	94%	91%	Site 2	Small	OL782345
26789	***Fusarium* sp.**	***F. sporotrichioides***	94%	91%	Site 2	Small	OL782341
26792	***F. transvaalense***	***F. sporotrichioides***	94%	91%	Site 2	Small	OL782351
26795	***Fusarium* sp.**	***F. sporotrichioides***	94%	91%	Site 2	Small	OL782343
27128	***F. transvaalense***	***F. brachygibbosum***	94%	93%	Site 2	Small	OL782337
27145	***Fusarium* sp.**	***F. brachygibbosum***	94%	93%	Site 2	Small	OL782336
27322	***F. transvaalense***	***F. sporotrichioides***	94%	91%	Site 2	Small	OL782350
27326	FSAMSC	*F. brachygibbosum*	93%	93%	Site 2	Small	OL782339
27530	FSAMSC	*F. brachygibbosum*	94%	94%	Site 1	Large	OL782354

Marked in bold are the isolates that produced contrasting nBLAST™ results between the two databases. All similarity percentages were below 97%.

**TABLE 4:** Nucleotide BLAST (nBLAST™) results from the *Fusarium* MLST and *FUSARIUM*-ID databases for the FIESC.

PPRI no.	*Fusarium* MLST	*FUSARIUM*-ID	*Fusarium* MLST similarity%	*FUSARIUM*-ID similarity%	Isolation site	Particle size	Accession number
24788	FIESC (10-a)	FIESC 10-a	99%	99%	Site 1	Small	OL782326
25048	FIESC (10-a)	FIESC 10-a	99%	99%	Site 1	Small	OL782327
25483	FIESC (10-a)	FIESC 10-a	99%	99%	Site 1	Large	OL782330
25486	FIESC (10-a)	FIESC 10-a	99%	99%	Site 1	Small	OL782332
25487	FIESC (10-a)	FIESC 10-a	99%	99%	Site 1	Small	OL782331
25490	FIESC (10-a)	FIESC 10-a	99%	99%	Site 1	Small	OL782328
25500	FIESC (9-b)	FIESC 9-b	96%	96%	Site 1	Large	OL782325
25505	FIESC (10-a)	FIESC 10-a	99%	99%	Site 1	Large	OL782329
26266	FIESC (9-b)	FIESC 9-b	100%	100%	Site 2	Large	OL782318
26299	FIESC (9-b)	FIESC 9-b	99%	99%	Site 2	Small	OL782321
26314	FIESC (9-b)	FIESC 9-b	100%	100%	Site 2	Small	OL782320
27406	***F. equiseti***	**FIESC 9-b**	100%	99%	Site 1	Large	OL782322
27424	FIESC (9-b)	FIESC 9-b	99%	99%	Site 1	Large	OL782324
27425	***F. equiseti***	**FIESC 5-d**	100%	99%	Site 1	Large	OL782317
27426	FIESC (9-b)	FIESC 9-b	100%	99%	Site 1	Large	OL782319
27523	FIESC (9-b)	FIESC 9-b	99%	99%	Site 1	Large	OL782323
28106	FIESC (28-a)	FIESC 28-a	99%	99%	Site 1	Large	OL782333

Marked in bold are the isolates that produced contrasting nBLAST™ results between the two databases. Marked in blue are percentage similarities lower than 97%.

**TABLE 5:** Nucleotide BLAST (nBLAST™) results from the *Fusarium* MLST and *FUSARIUM*-ID databases for the FFSC.

PPRI no.	*Fusarium* MLST	*FUSARIUM*-ID	*Fusarium* MLST similarity%	*FUSARIUM*-ID similarity%	Isolation site	Particle size	Accession number
26239	*F. fujikuroi*	GFSC	99%	98%	Site 2	Small	OL782562
26300	GFSC*	GFSC	98%	98%	Site 2	Small	OL782567
26321	GFSC	GFSC	98%	98%	Site 2	Small	OL782566
26329	*F. fujikuroi*	GFSC	98%	98%	Site 2	Small	OL782563
27327	**GFSC**	***F. andiyazi***	97%	96%	Site 2	Small	OL782569
27410	**GFSC**	***F. andiyazi***	97%	96%	Site 1	Large	OL782568
27432	**GFSC**	***F. andiyazi***	97%	97%	Site 2	Small	OL782570
27526	GFSC	GFSC	98%	98%	Site 1	Large	OL782564
27540	GFSC	GFSC	98%	98%	Site 1	Large	OL782565

*Gibberella fujikuroi species complex (GFSC) (Geiser et al., 2013). Marked in bold are the isolates that produced contrasting nBLAST™ results between the two databases. Marked in blue are percentage similarities lower than 97%.

**TABLE 6:** Nucleotide BLAST (nBLAST™) results from the *Fusarium* MLST and *FUSARIUM*-ID databases for the FCSC.

PPRI no.	*Fusarium* MLST	*FUSARIUM*-ID	*Fusarium* MLST similarity%	*FUSARIUM*-ID similarity%	Isolation site	Particle size	Accession number
26282	FCSC (1-m)	FCSC (1-m)	97%	97%	Site 2	Small	OL782355
27412	FCSC (5-a)	FCSC (2-a)	100%	99%	Site 1	Large	OL782357
27417	FCSC (5-a)	FCSC (2-a)	100%	99%	Site 1	Large	OL782358
27419	FCSC (5-a)	FCSC (2-a)	99%	99%	Site 1	Large	OL782359
27506	*F. cf. chlamydosporum*	FCSC (2-a)	99%	99%	Site 1	Large	OL782360
27516	FCSC (5-a)	FCSC (2-a)	99%	99%	Site 1	Large	OL782356

**TABLE 7:** Nucleotide BLAST (nBLAST™) results from the *Fusarium* MLST and *FUSARIUM*-ID databases for the FSSC.

PPRI no.	*Fusarium* MLST	*FUSARIUM*-ID	*Fusarium* MLST similarity%	*FUSARIUM*-ID similarity%	Isolation site	Particle size	Accession number
25492	FSSC (5-c)	FSSC (5-d)	100%	100%	Site 1	Small	OL782571
25506	FSSC	FSSC (5-i)	100%	100%	Site 1	Small	OL782573
25509	FSSC (5-c)	FSSC (5-d)	100%	100%	Site 1	Small	OL782572

### Phylogenetic analysis

Phylogenetic analyses for the individual species complexes were based on nBLAST™ species complex identification and isolation frequency resulting in five phylogenetic analyses. Phylogenetic analyses using the aligned sequences for the different datasets were based on Maximum Likelihood (ML) and Bayesian Inference (BI). The ML and BI phylogenetic trees were topologically congruent, and the presented topology is based on the ML tree results. The presented phylogenetic trees (Figures 5–8) highlight the clades in which the PPRI isolates are resolved. The corresponding supplementary figures showing the full phylogenetic trees are indicated at each of the respective phylogenetic figures. Due to multiple phylogenetically identical isolates found in the *F. oxysporum* species complex (FOSC) (sensu Smith and Swingle) (based on nBLAST™ analyses) the resulting FOSC analysis was performed with 53 of the original 199 isolates identified as belonging to the FOSC. Members identified among the *F. sambucinum* species complex (FSAMSC) (Fückel) (sensu stricto), the *F. incarnatum-equiseti* species complex (FIESC) [(Desm.) (Corda) Sacc. 1886] and the *F. fujikuroi* species complex (FFSC) [(Sawada) S. Ito in Ito and K. Kimura] were evaluated using separate datasets (Table 1). The three species complexes, respectively comprising less than 2% of the total 257 isolates were analysed with reference isolates from various publications that identified similar *Fusarium* species complex isolates from natural environments (Table 1). These species complexes include the *F. chlamydosporum* species complex (FCSC) (Wollenw. and Reinking), the *F. solani* species complex (FSSC) [(Mart.) Sacc. 1881] and the *F. nisikadoi* species complex (FNSC) (Aoki and Nirenberg, 1999).

The generated *tef-1*α sequences and reference isolates were aligned using MAFFT (Katoh and Standley, 2013) and manually edited where necessary. For the ML and BI analyses the parameters were determined using the online platform SMS: Smart Model Selection (Lefort et al., 2017) with the Akaike information criterion. The recommended substitution models used were GTR + G for the FOSC and the FFSC and GTR + G + I for the FSAMSC, the FIESC and the minor species complex analyses (Table 1). The ML analyses were determined using the RAxML (version 7.2.8) plug-in (Thompson et al., 1997) in Geneious 8.1.9 (Kearse et al., 2012). The clade stability was assessed by bootstrap support (BS) using 1 000 bootstrap replicates and default parameters. The BI analysis was performed through the online portal CIPRES^3^ (Miller et al., 2010) using the MRBayes v. 3.2.5 on XSEDE using four incrementally heated MCMC chains. The chain length was set to run 10 million generations and tree sampling was done for every 1 000 trees. The burn-in length was set to 100 000. The BI was used to calculate Posterior probability (PP) values for consensus nodes. Only statistical support values ≥0.98 for PP values and ≥70% for ML-BS were accepted. The topology used for each dataset is based on the BI. The resulting phylogenetic trees were plotted and visualised using FigTree (version 1.4.4)^4^ (Rambaut, 2013).

The respective phylogenetic trees were rooted using outgroup isolates used in the referenced datasets (Table 1). The following outgroup isolates were selected for the respective species complex analyses: *Fusarium* sp. (NRRL 25184) for the FOSC analysis, *F. nelsonii* (NRRL 13338) for the FSAMSC analysis, *F. oxysporum* (NRRL 22902) for the FFSC analysis, *F. concolor* (NRRL 13459) for the FIESC analysis and *F. lateritium* (NRRL 52786 and NRRL 25122) for the minor species complex analysis. The reference sequences as well as the sequence data for the outgroup species were mined from the NCBI Genbank database. The data presented in this study are deposited in the Genbank repository, accession numbers OL782317–OL782573.

## Results

### Representative morphological characters

**FIGURE 2:**
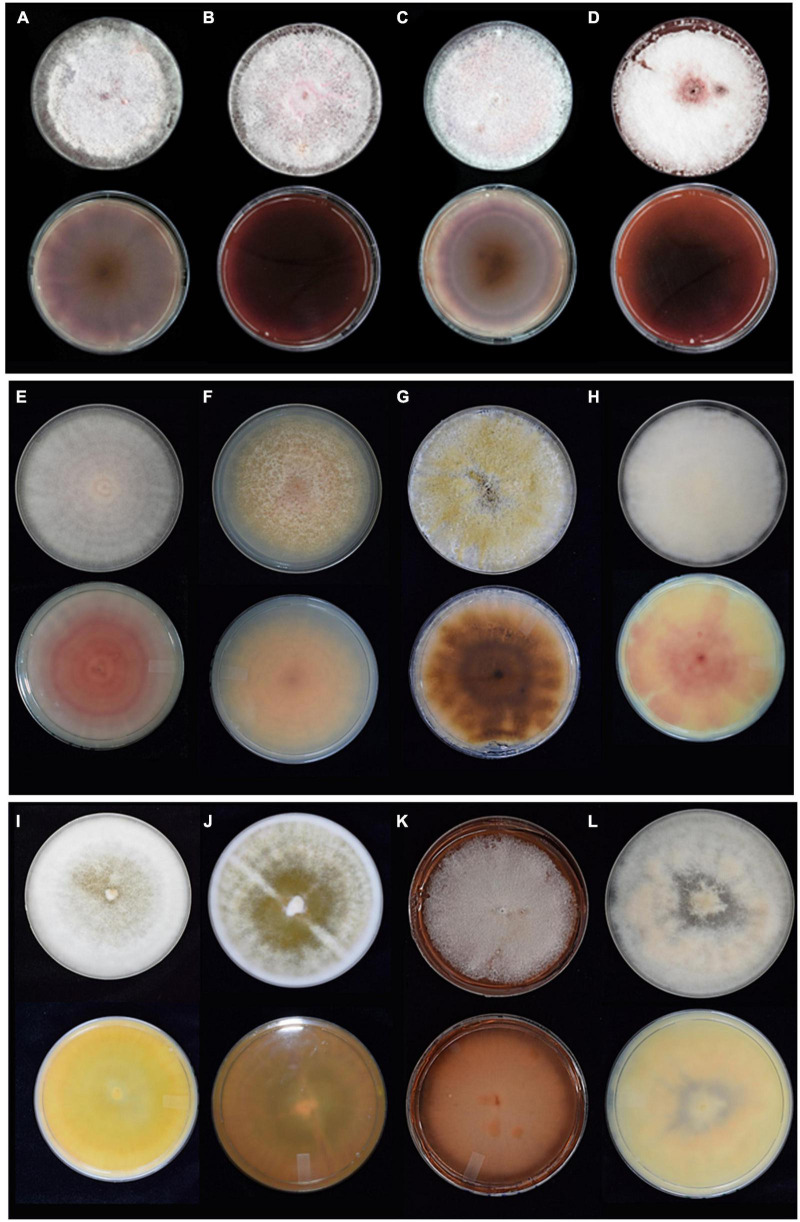
The gross morphology growth (top row) and colony pigmentation (bottom row) for representative isolates of the different species complexes cultured on PDA for 10 days. **(A)** FOSC (PPRI 25045), **(B)** FOSC (PPRI 25046), **(C)** FOSC (PPRI 25504), **(D)** FOSC (PPRI 25049), **(E)** FFSC (PPRI 27410), **(F)** FFSC (PPRI 27540), **(G)** FSSC (PPRI 25506), **(H)** FCSC (PPRI 26282), **(I)** FSAMSC (PPRI 26792), **(J)** FSAMSC (PPRI 27530), **(K)** FIESC (PPRI 25500), and **(L)** FIESC (PPRI 27406).

**FIGURE 3:**
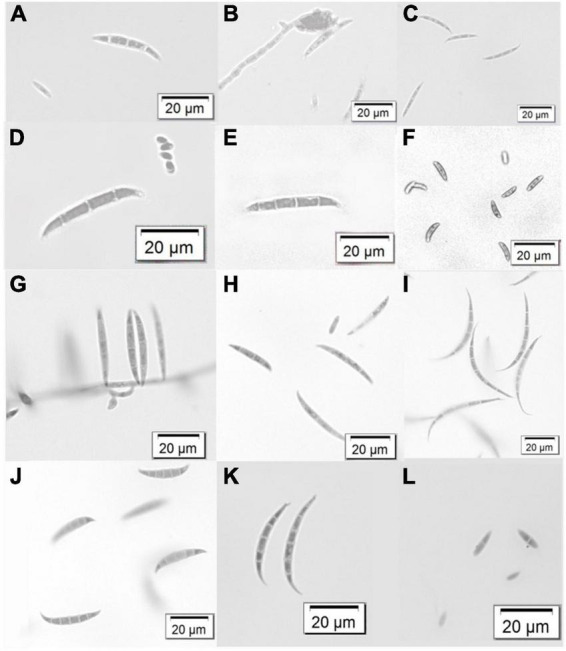
Variation in macroconidium structures and some microconidia of the identified *Fusarium* species complexes from the GGHNP. FOSC (PPRI 26304) **(A–C)**, FSSC (PPRI 25492) **(D,E)**, FFSC (PPRI 27410) **(F–H)**, FIESC (PPRI 27406) **(I)**, FSAMSC (PPRI 27530) **(J)**, FCSC (PPRI 26282) **(K,L)**. Images were visualised using a light microscope at 40X magnification.

The gross colony morphology and pigmentation is shown in Figure 2 for the different species complexes after culturing on PDA. The FOSC’s characteristic pale violet and dark magenta pigments produced on the agar was evident (A-D). The different pigments or colourations were observed for the FFSC (E and F), the FSSC (G), the FCSC (H), FSAMSC (I and J), and the FIESC (K and L). The morphological characters of the FOSC, FSSC, FIESC, FCSC, FFSC, and FSAMSC identified from the GGHNP were studied to provide an overview of the common morphological characters (Figure 3). The macroconidia for the FOSC isolate PPRI 26304 were 3-septate and slightly curved, with the microconidia showing 0-septa (A and C). The microconidia were produced on a short monophialide with a false head (B). The macroconidia produced by the FSSC (PPRI 25492) on CLA (D and E) were 3- to 4-septate with a blunt and rounded apical cell morphology. The FFSC (PPRI 27410) shows 3-septa macroconidia produced on CLA (G and H) with a single microconidium amongst the macroconidia in H and multiple microconidia in F that appear uniform in size. The FIESC (PPRI 27406) macroconidia were long, slender and 5-septate showing an elongated foot shape at the basal end and an elongated whip-like appearance at the apical cell (I). The FSAMSC (PPRI 27530) displayed its characteristic short 5-septate macroconidia produced on CLA (J). The apical cell appears to be pointed with the basal cell being foot shaped. Microconidia were not present on the CLA, as expected. The FCSC (PPRI 26282) isolate produced 5-septate macroconidia on CLA (K). The macroconidia, which are not commonly produced by FCSC, appear to have a foot shaped basal cell morphology. The FCSC microconidia produced on CLA were 0- to 1-septate (L). The morphological characters were consistent with the characters per species complex as identified using Leslie and Summerell (2006).

**FIGURE 4:**
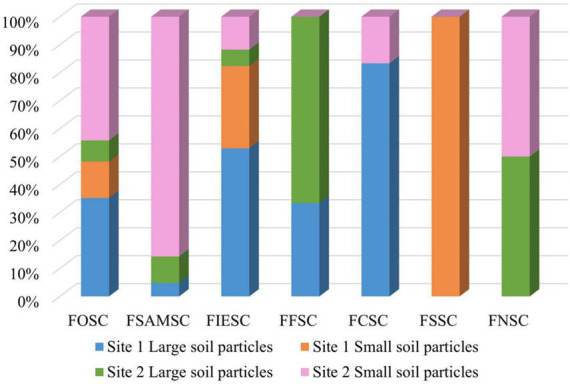
The number of isolates obtained per species complex between the two sampling sites from the respective soil particle sizes.

Relatively similar numbers of isolates were obtained from the two sampled sites. Site 1 contributed 122 isolates (47.5%) and site 2 contributed 135 isolates (52.5%). Most of the FOSC isolates (44%) were found from site 2’s small soil particles followed by site 1’s large soil particles (35%) (Figure 4). The rest of the FOSC isolates were isolated from site 1’s small soil particles (13%) and site 2’s large soil particles (8%). Of the FSAMSC isolates 86% came from site 2’s small soil particles and 9% from the large particles of site 2. Only a single (5%) FSAMSC isolate was obtained from the large particles of site 1, with no isolates identified from site 1’s small soil particles. Isolates identified as belonging to the FIESC were found amongst both sites and soil particle sizes with 53% from site 1’s large particles, 29% from site 1’s small soil particles. With 6% and 12% of the FIESC isolates from site 2’s large and small soil particles, respectively. The FCSC isolates were obtained as 83% from site 1’s large soil particles and only one isolate (17%) from site 2’s small soil particles. The three (100%) FSSC isolates were only found in site 1 from small soil particles and the FNSC were found in site 2, with one isolate (50%) obtained from small and the other (50%) from large soil particles.

**FIGURE 5:**
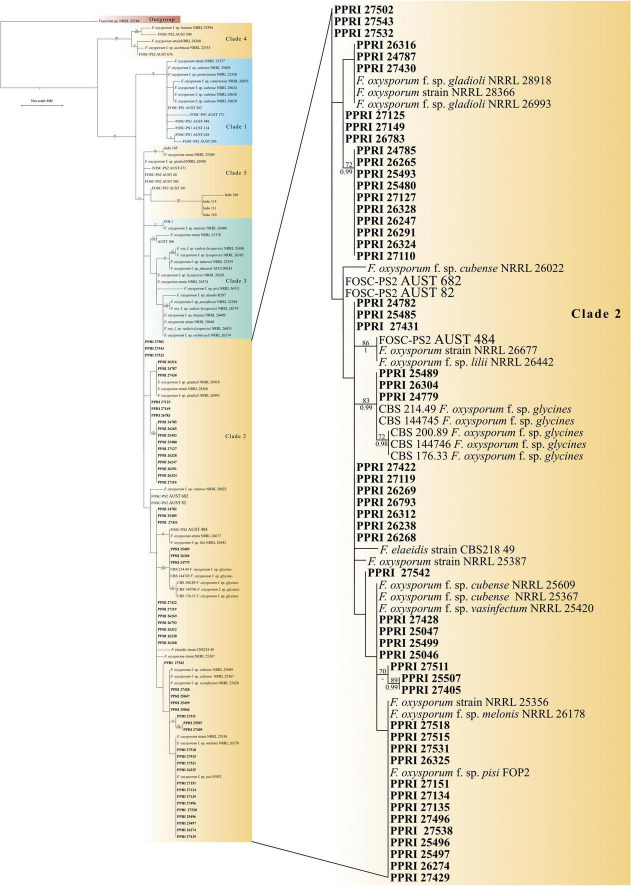
Phylogenetic tree of the FOSC dataset inferred from the *tef-1*α gene region for 123 taxa. Isolates from the GGHNP are indicated by PPRI number and are marked in bold. Branch support values are indicated as ML-BS/PP values (>0.98/>70%) above the branches at the corresponding nodes.

**FIGURE 6:**
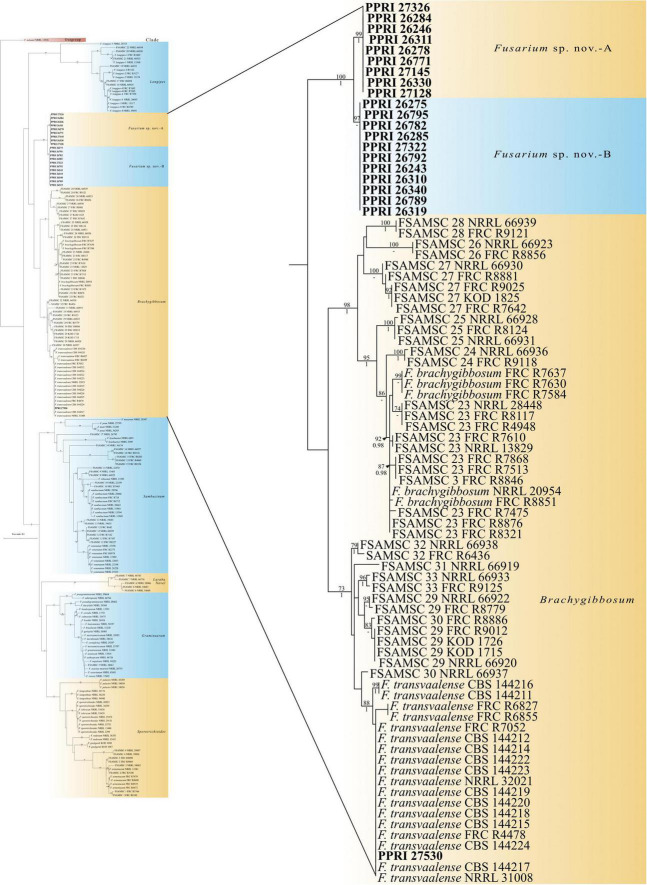
Phylogenetic tree of the FSAMSC dataset inferred from the *tef-1*α gene region for 204 taxa. Isolates from the GGHNP are indicated by PPRI number and are marked in bold. Branch support values are indicated as ML-BS/PP values (>0.98/>70%) above the branches at the corresponding nodes.

**FIGURE 7:**
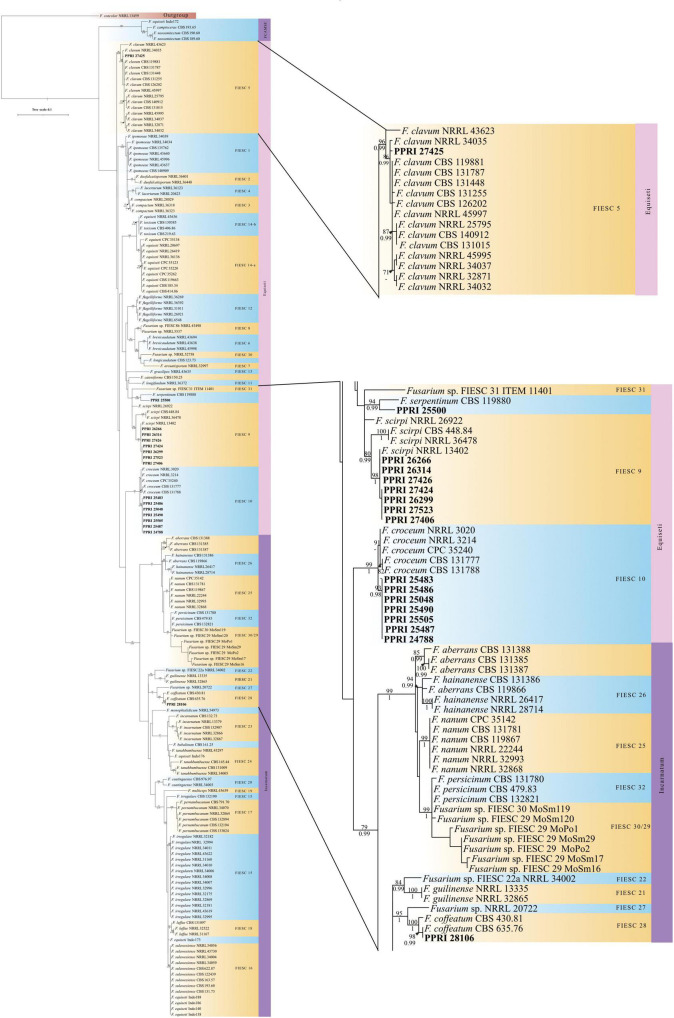
Phylogenetic tree of the FIESC dataset inferred from the *tef-1*α gene region for 175 taxa. Isolates from the GGHNP are indicated by PPRI number and are marked in bold. Branch support values are indicated as ML-BS/PP values (>0.98/>70%) above the branches at the corresponding nodes.

**FIGURE 8:**
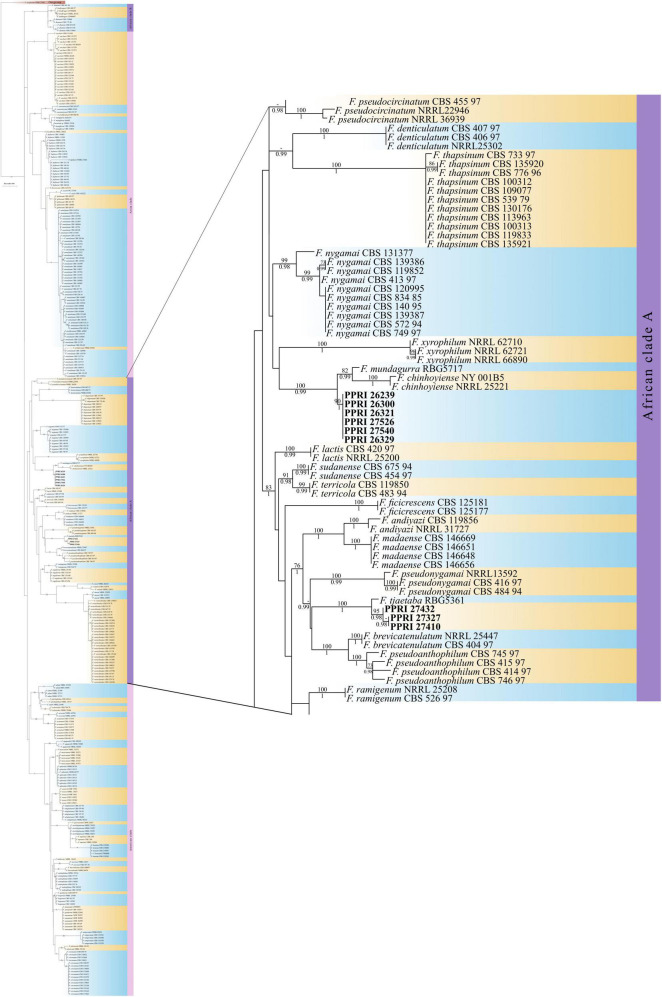
Phylogenetic tree of the FFSC dataset inferred from the *tef-1*α gene region for 359 taxa. Isolates from the GGHNP are indicated by PPRI number and are marked in bold. Branch support values are indicated as ML-BS/PP values (>0.98/>70%) above the branches at the corresponding nodes.

### Phylogenetic analyses

#### FOSC

Among the 199 FOSC isolates, the multilocus sequence type (MLST), or haplotypes, were determined and some species-specific identifications were made only against the *Fusarium*-MLST database (Table 2). The MLST types refer to clinically important *Fusarium* species represented as a haplotype code or a number. From the 199 FOSC isolates, there were 60 isolates identified with identical haplotypes by both the *Fusarium* MLST and the *FUSARIUM*-ID databases’ nBLAST™ identities. Fourteen of these similarities are indicated by the grey highlighted rows in Table 2. A total of six haplotypes were identified among the FOSC isolates using both databases. From these 61 isolates, 50.82% were identified as haplotype 18 followed by haplotype 191 contributing 37.70% and haplotype 47 at 6.56%. Haplotype 179 was identified as 3.28% and both haplotype, 29 and 222 were identified as 1.64% of the isolates. Twenty-nine of the 199 FOSC isolates had conflicting haplotypes between the two databases. nBLAST™ data for all 199 isolates are not shown. Nine of these conflicting isolates are indicated in bold in Table 2.

The FOSC phylogeny resolved to form five distinguished clades (Figure 5; Supplementary Figure 1). All 53 of the FOSC PPRI isolates selected for the phylogenetic analysis grouped within the larger clade 2 as demarcated by previous studies (O’Donnell et al., 1998b,^2004^; Laurence et al., 2014). Within clade 2 the PPRI isolates formed multiple subclades that were mostly unresolved or produced low resolution. Only one subclade comprising three PPRI isolates and five *F. oxysporum* f. sp. *glycines* reference isolates was highly resolved (BPP = 0.99; ML-BS = 83%).

#### FSAMSC

The twenty-one FSAMSC isolates, produced the lowest identification resolution when compared to the other six species complexes. The nBLAST™ similarity percentages were all below 94% similarity and most of the isolates produced conflicting nBLAST™ identifications against the respective databases (Table 3). Species-specific identifications were made for six isolates linking to the *F. transvaalense* against the *Fusarium*-MLST database only and all the identifications made against the *FUSARIUM*-ID database were reported as either *F. sporotrichioides* or as *F*. *brachygibbosum*. The phylogenetic analysis based only on the *tef-1*α supports the six monophyletic clades designated previously (Laraba et al., 2021), an additional, strongly supported, seventh clade is revealed through the current study. This novel clade clusters basal to the designated *Brachygibbosum* clade and comprised of twenty PPRI isolates collected predominantly from site 2 of the GGHNP (Figure 4). This novel FSAMSC clade formed two closely related subclades, with the split strongly supported (BPP = 1; ML-BS = 100%). These two subclades represent putatively novel unnamed species which will further be referred to as *Fusarium* sp. nov.-A and *Fusarium* sp. nov.-B as indicated in Figure 6; Supplementary Figure 2. *Fusarium* sp. nov.-A and *Fusarium* sp. nov.-B is represented by nine and 11 isolates, respectively. A single isolate from the current study, PPRI 27530, nested with *F. transvaalense* isolates that grouped within the larger *Brachygibbosum* clade of the FSAMSC analysis (Figure 6). Although this isolate did not produce a nBLAST™ identification linking to *F. transvaalense* the phylogenetic grouping supports its relationship with this recently described species (Sandoval-Denis et al., 2018).

#### FIESC

The FIESC isolates were the third most frequently isolated and between four to five MLST types were identified (against both databases) among the seventeen FIESC isolates (Table 4). The FIESC PPRI isolates obtained showed seven of these identified as FIESC MLST 10-a and another seven as FIESC MLST 9-b. All the nBLAST™ similarity percentages were above 99% except for isolate PPRI 25500, identified as FIESC MLST 9-b, which produced a similarity percentage of 96% against both databases.

The phylogeny for the FIESC analysis resolved the ingroup taxa into the three main clades (Figure 7; Supplementary Figure 3) also found in Xia et al. (2019). The first clade demarcated as the *F. camptoceras* species complex (FCAMSC) (BPP = -; ML-BS = 100%), was resolved as the sister clade to the larger second clade, the *Equiseti* clade. The third of the larger clades, demarcated as the *Incarnatum* clade (BPP = 0.99; ML-BS =), grouped basal to the *Equiseti* clade.

The FIESC PPRI isolates from the current study grouped independently in five subclades, with sixteen isolates resolved within subclades of the larger *Equiseti* clade and only one isolate resolved within the larger *Incarnatum* clade. There were seven isolates resolved as being closely related to *F. croceum* (FIESC 10) (BPP = 1; ML-BS = 99%) and another seven resolved as *F. scirpi* (FIESC 9) (BPP = 0.99; ML-BS = 80) that corresponds to the nBLAST™ identifications. The three remaining PPRI isolates are represented by single strain isolates/lineages. The isolate PPRI 27425 resolved with the *F. clavum* subclade (FIESC 5) (BPP = 0.99; ML-BS = 96%) and PPRI 25500 resolved with the *F. serpentinum* subclade represented by a single strain (no FIESC MLST indicated) (BPP = 0.99; ML-BS = 94%), both subclades were grouped within the larger *Equiseti* clade. PPRI 25500 had a low similarity percentage (96.14%) and that the nBLAST identity linked to FIESC MLST 9-b (against both databases), yet this isolate did not group within the *F. scirpi* (FIESC MLST 9) clade and instead grouped with the recently described *F. serpentinum* ex-type culture (CBS 119880) that is closely related to *F. scirpi*. PPRI 28106, the only isolate that grouped within the larger *Incarnatum* clade, resolved within the *F. coffeatum* subclade (FIESC 28) (BPP = 1; ML-BS = 100%).

#### FFSC

There were nine PPRI isolates identified as belonging to the FFSC (Table 5). From these nine isolates three isolates produced similarity percentages below 98%. These three isolates also produced conflicting identifications against the respective databases, with the *FUSARIUM*-ID database indicating the species level identification as *F*. *andiyazi* (Marasas, Rheeder, Lampr., K. A. Zeller and J.F. Leslie) and the *Fusarium*-MLST database linking to either the FFSC or just *Fusarium* sp. (Table 5).

All the FFSC PPRI isolates from the current study were phylogenetically resolved in the larger African clade (A) (Figure 8; Supplementary Figure 4) (Yilmaz et al., 2021). The six FFSC isolates were phylogenetically closely related to *F. chinhoyiense* isolates and a single *F. mundagurra* isolate (RBG5717). This close relationship was highly supported (BPP = 0.99; ML-BS = 100%). *F. chinhoyiense* is a recently described phylo-species, obtained from Zimbabwean and South African soils (Yilmaz et al., 2021), that is closely related to *F. mundagurra* isolates, obtained from uncultivated Australian soils (Laurence et al., 2016). The six PPRI isolates formed a distinct sister clade to the above-mentioned reference isolates indicating a possible new closely related species. The three isolates that linked with *F. andiyazi*, based on nBLAST™ results, did not group phylogenetically with *F. andiyazi* reference isolates but formed a well-supported (BPP = 1; ML-BS = 100%) sister clade to a single *F. tjaetaba* (NRRL 66243) reference isolate, which were also described from natural ecosystems in Australia (Laurence et al., 2016). This close yet distinguished relationship may indicate another possible new species found in the GGHNP belonging to the FFSC.

### Minor species complex analysis

The minor species complex analyses include six FCSC, three FSSC and two FNSC PPRI isolates obtained from the current study. The six FCSC isolates obtained produced varying identifications against the respective databases. PPRI 26282, was the only isolate to produce an identical MLST against both databases and identified as FCSC MLST 1-m. The nBLAST™ result for the remaining FCSC isolates were identical to each other but differed between the respective databases as either FCSC MLST 5-a and MLST 2-a (Table 6). The *F. chlamydosporum* clade grouped basal to the *F. aywerte* clade in the minor species complex phylogenetic analysis. The *F. chlamydosporum* clade comprised seven reference isolates identified as belonging to the FCSC and six undescribed PPRI isolates from the current study. The monophyly of this clade was strongly supported (BPP = 1; ML-BS = 98%), supporting the nBLAST™ identifications that included the six PPRI isolates in the FCSC. The isolate, PPRI 26282, that linked to FCSC MLST 1-m resolved with reference isolate GQ505413 with low support (BPP = none; ML-BS = 77). Reference isolate GQ505413 was identified as FCSC MLST 1-m supporting the identification of PPRI 26282 as MLST 1-m (O’Donnell et al., 2009).

The three FSSC isolates produced two isolates (PPRI 25492 and PPRI 25509) that identified as MLST 5-c against the *Fusarium* MLST database and as MLST 5-d against the *FUSARIUM*-ID database (Table 7). The third FSSC isolate (PPRI 25506) identified as MLST 5-i on the *FUSARIUM*-ID database and grouped with the reference isolate identified as FSSC MLST 5-i (DQ246922). The minor species complex tree shows the three FSSC isolates forming a well-supported clade with the FSSC reference isolates, supporting their identification as members of the FSSC. The grouping of the *F. solani* clade was fully resolved (BPP = 1; ML-BS = 100%).

**FIGURE 9:**
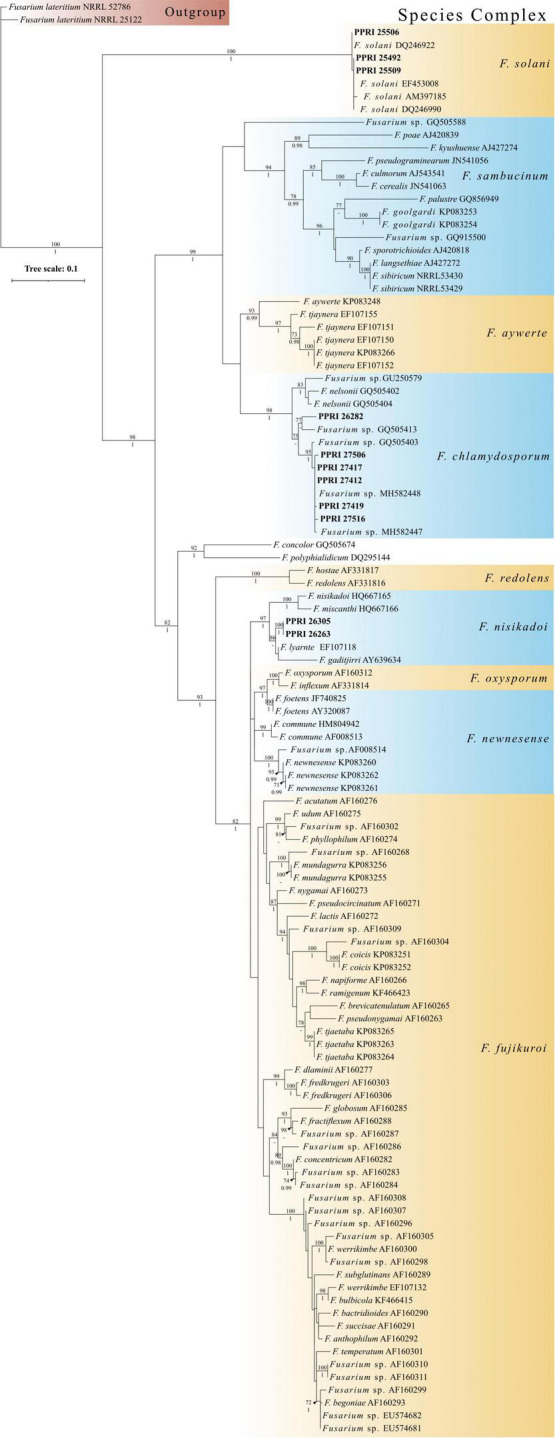
Phylogenetic tree of the minor species complex dataset inferred from the *tef-1*α gene region for 113 taxa. Isolates from the GGHNP are indicated by PPRI number and are marked in bold. Branch support values are indicated as ML-BS/PP values (>0.98/>70%) above the branches at the corresponding nodes.

The two FNSC PPRI isolates (Table 8) produced high percentage similarities against the *Fusarium*-MLST database but low percentages against the *FUSARIUM*-ID database. The nBLAST™ results indicated to the species level identifications as *F. lyarnte* and *F. gaditjirri*. From the minor species complex (Figure 9; Supplementary Figure 5) phylogenetic analysis, the two PPRI isolates grouped with strong support with the FNSC reference isolates to form the *F. niskikadoi* clade. The two FNSC PPRI isolates’ relationship to the *F. lyarnte* and *F. gaditjirri* (EF107118 and AY639634) reference isolates was resolved by BS support of 86%. The FNSC is a known sister clade (Baayen et al., 2001) to the FOSC and this grouping also exists in the current minor species complex analysis (Figure 9).

**TABLE 8:** Nucleotide BLAST (nBLAST™) results from the *Fusarium* MLST and *FUSARIUM*-ID databases for the FNSC.

PPRI no.	*Fusarium* MLST	*FUSARIUM*-ID	*Fusarium* MLST similarity%	*FUSARIUM*-ID similarity%	Isolation site	Particle size	Accession number
26263	***F. lyarnte***	***F. gaditjirri***	98%	96%	Site 2	Large	OL782560
26305	***F. lyarnte***	***F. gaditjirri***	98%	96%	Site 2	Small	OL782561

Marked in bold are the isolates that produced contrasting nBLAST™ results between the two databases. Marked in blue are percentage similarities lower than 97%.

## Discussion

The diversity of soil-borne *Fusarium* isolates sampled from the GGHNP were analysed to further contribute to research on South African soils linked to presumed non-pathogenic strains of *Fusarium* (Marasas et al., 1988; Jeschke et al., 1990; Rheeder and Marasas, 1998; Mojela, 2017; Jacobs et al., 2018; Sandoval-Denis et al., 2018; Mavhunga, 2021). The use of the *tef1-*α gene region for molecular identification of *Fusarium* diversity found in the GGHNP was a key instrument to achieve this. The undisturbed and semi-disturbed soils of the grassland biome have proven to harbour a high level of microbial diversity, including fungi like *Fusarium*. The grassland biome is of importance as many households in South Africa rely on subsistence farming that depends on the health of the grassland biome. This study found seven *Fusarium* species complexes with varying levels of diversity and relationships to pathogenic isolates. The possibility of novel species found within the FSAMSC and the FFSC, for which the pathogenicity is still unknown, may have an influence on agricultural systems in South Africa. This study builds on studies on diversity of *Fusarium* members found in natural ecosystems, such as the grassland biome, with low anthropogenic activity in South Africa, aimed at surveying the diversity of *Fusarium* found in South African soil.

Previous studies on South African soils and plant debris found the FOSC to be most dominant (Marasas et al., 1988; Jeschke et al., 1990; Rheeder and Marasas, 1998; Mavhunga, 2021) followed by the FSSC and the FIESC. Marasas et al. (1988) included the GGHNP as one of 29 sites sampled in South Africa from which ten different *Fusarium* species were identified based solely on morphological characterisation. The GGHNP was one of five sites that produced high levels of species diversity (i.e., ten different species) from 56 GGHNP isolates. There are three species complexes identified from the GGHNP that agree with the findings of Marasas et al. (1988), the FOSC, FSAMSC and the FIESC. When only looking at the GGHNP isolates from the Marasas et al. (1988) study, *F. sambucinum* (FSAMSC) and *F. equiseti* (FIESC) were the second and third most frequently isolated species, recognising that these morpho-species may have hidden further diversity. Our study further identified members belonging to the FCSC and the FSSC, that were not identified in the Marasas et al. (1988) study. This trend extrapolated to comparisons with the Balmas et al. (2010) study with only three FSSC isolates obtained, these were isolated least frequently. All the species complexes were isolated in varying frequencies from both large and small soil particles except for the FSSC, which were only isolated from small soil particles of site 1. This may be why Marasas et al. (1988) did not identify any FSSC from the GGHNP as they only sampled plant debris. Jeschke et al. (1990) suggested that the measure of diversity between the sampling techniques, such as the collected sample material used, may be subjective and can be influenced by the mode of survival of the species isolated.

The FOSC present a very complicated taxonomy. Between three (O’Donnell et al., 1998b), five (Laurence et al., 2012) and eight (Lombard et al., 2019a) reoccurring clades have been reported and extensively studied in this species complex (Achari et al., 2020). Initially clades one, two and three represented several morphologically cryptic species and a fourth clade was introduced through the inclusion of clinically important FOSC isolates (O’Donnell et al., 2004). Through the incorporation of Genealogical Concordance Phylogenetic Species Recognition (GCPSR) studies the recognised phylogenetic species ranged from two (Laurence et al., 2014) to twenty-one (Lombard et al., 2019a). The study by Laurence et al. (2014) implemented eight gene regions using GCPSR studies and determined seventeen independent evolutionary lineages among members of the FOSC, which were congregated to indicate two phylogenetic species (PS) demarcated as PS1 and PS2. The species present in clade 1 formed PS1 and clades 2, 3, 4, and 5 are regarded as PS2. None of the GGHNP isolates grouped with PS1 isolates. Clade 5 housed FOSC PS2 reference isolates from Australia (Laurence et al., 2012) as well as Indonesian isolates (Maryani et al., 2019a), indicating a wide biogeographical scope. The FOSC are a community dominant group with a high level of genetic diversity, found in semi- and un-disturbed areas, and this contributes to the efforts made in resolving the FOSC phylogeny (Nash and Snyder, 1965; Laurence et al., 2012; Mavhunga, 2021).

The percentage of FOSC and FIESC isolates obtained were among the top featured species complexes. Studies on South African grassland soils from lower altitudes, also found the FOSC and the FIESC to be highly diverse (Mojela, 2017; Mavhunga, 2021). The FOSC population differed in species diversity between the two sampled sites. This corroborates the findings from French soils, where the FOSC had a varied diversity within the same sampling location and differed in variety when compared with other sampling locations (Edel et al., 2001). For instance, FOSC haplotype 18 was isolated more frequently from site 2 and FOSC haplotype 191 was isolated more frequently from site 1, although both haplotypes were present in both the respective sites. Studying the distribution patterns of such species complexes may indicate host-range, climate-range or the influence of anthropogenic distribution (Summerell et al., 2010). The current study contributes to this goal (Laurence et al., 2012, 2016) of identifying the range of *Fusarium* species distribution in South Africa.

The GGHNP contain a variety of wild *Protea* species that are of high conservation value (Mabunda, 2011) and it has been found that *F. oxysporum* (species not defined) is capable of infecting at least six *Protea* cultivars causing Fusarium Wilt (Swart et al., 1999). This supports the cause for concern as the FOSC were most frequently isolated from the GGHNP soils. As emphasised by Laurence et al. and Swart et al. (1999), by evaluating the phylogenetic distance between non-cultivated and agricultural strains it could indicate a climatic or edaphic pattern which is suggested to contribute to the diversity and distribution of certain species complexes, such as the FOSC. With the grassland biome used for a wide range of commercial and agricultural uses (forestry, animal grazing, mining, commercial and subsistence crop production), the remaining undisturbed hectares demands protection and conservation (Mabunda, 2011; Mavhunga, 2021).

The FSAMSC PPRI grouping most likely represents more than one novel *Fusarium* species belonging to the FSAMSC that are related to *F*. *transvaalense* and *F. brachygibbosum*. *Fusarium sporotrichioides* reference isolates were included (Xia et al., 2019) in the current study and none of the PPRI isolates that produced a nBLAST™ identification (against either database) as *F*. *sporotrichioides* grouped within the *Sporotrichioides* clade. Thus, there is a very strong possibility of new species discovered from the GGHNP. The discovery of novel species based on phylogenetic analysis is occurring more frequently, especially in studies on undisturbed soils, and it has been found that novel species tend to be geographically restricted to certain sites (Balmas et al., 2010; Laurence et al., 2016; Summerell, 2019). In order to confirm this, the inclusion of additional gene regions such as the *RPB1* and *RPB2* gene regions are needed (O’Donnell et al., 2013, 2015).

O’Donnell et al. (2009) implemented the informal haplotype naming system, originally developed by Chang et al. (2006) to facilitate communication of clinically important species that lack Latin binomials. These haplotypes are referred to as MLST types. Previously only 28 FIESC have been assigned Latin binomials (O’Donnell et al., 2009), with many more recently being formally named (Maryani et al., 2019b; Santos et al., 2019; Wang et al., 2019; Xia et al., 2019). Some conflicts arise as the same MLST designation (MLST 29 and 30) was used in different studies (O’Donnell et al., 2012; Torbati et al., 2019) that was later designated as MLST 33 by Wang et al. (2019). As only some of the FIESC isolates related to mycotic infections have accepted Latin binomials it makes accurate reporting by clinicians and veterinarians difficult (O’Donnell et al., 2009; Xia et al., 2019). There are currently 38 recognised lineages found within the FIESC (Lima et al., 2021) that group phylogenetically within either the *Equiseti* or the *Incarnatum* clades (Villani et al., 2019). The phylogenetic species found in the *Equiseti* clade have been found to exhibit a cosmopolitan distribution and the *Incarnatum* clade predominantly includes isolates found in temperate regions (Ramdial et al., 2017; Lima et al., 2021). The FIESC MLST 9 (types 9-a, 9-b and 9-c) has a Latin binomial assigned as *F*. *scirpi* and the FIESC MLST 10 assigned as *F. croceum* (O’Donnell et al., 2009; Xia et al., 2019). The seven PPRI isolates, linked to FIESC MLST 10, grouped highly supported but basal to the *F. croceum* reference isolates indicating possible new species. The *F. croceum* ex-type was recently described by Xia et al. (2019) and further morphological and phylogenetic analysis with the inclusion of additional gene regions would be required to confirm the relation to *F. croceum*. The other seven PPRI isolates that linked to FIESC MLST 9 grouped with *F. scirpi* reference isolates. An early study on *F. scirpi* isolates showed it to be common in arid and semi-arid regions in South Africa with isolates obtained from seed pods, roots and plant debris from soil (Burgess et al., 1985). *F. scirpi* related isolates have also been reported from undisturbed natural soils from South Africa (Jacobs et al., 2018). The only FIESC isolate (PPRI 28106) from the current study that grouped within the larger *Incarnatum* clade was phylogenetically resolved as *F. coffeatum*. FIESC MLST 28 was identified to species level and named as *F. coffeatum* (Lombard et al., 2019b) and the morphological characters for the ex-type culture were evaluated and confirmed as *F. coffeatum* (Xia et al., 2019). A single isolate, PPRI 27425, nested within the *F. clavum* clade with nBLAST™ identifications supported by phylogenetic grouping. *F. clavum* was demarcated as FIESC MLST 5 and the nBLAST identity linked to FIESC MLST 5-d. Isolates phylogenetically closely related to FIESC MLST 5 reference isolates were associated with the death of cattle feeding on infected kikuyu grass (Botha et al., 2014; Jacobs et al., 2018). It is important to consider the ecology of each species as it contributes significantly to its description (Burgess et al., 1985).

The phylogenetic analysis on phytopathogenic members of the *F*. *fujikuroi* species complex (FFSC) (published under the GFSC name) by O’Donnell et al. (1998a) grouped members based on the biogeographic origin that was consistent with clade formation. These phylogenetic clades were designated as the African, American, and Asian clades. This hypothesis by O’Donnell et al. (1998a) is inconsistent with species falling within a certain geographically based clade, although being associated with hosts from a different geographical origin (Rheeder and Nelson, 1996; Aoki and Nirenberg, 1999; Bentley et al., 2007). The FFSC member known as *F*. *verticillioides*, for instance, falls within the African clade, although its most common host association is maize, of which the earliest ancestor is teosinte that originates from the Americas and not Africa (Steenkamp et al., 2001; Summerell et al., 2010). The FFSC isolates from the current study grouped within the African clade demarcated by O’Donnell et al., 1998a or within the African (A) clade as demarcated by Sandoval-Denis et al. (2018) and Yilmaz et al. (2021). The FFSC PPRI isolates could confidently be regarded as belonging to the FFSC, with three isolates regarded as new sister species to *F. tjaetaba* members and the remaining six isolates representing a new phylo-species closely related to *F. mundagurra* and *F. chinhoyiense* species. The FFSC is well represented in the *FUSARIUM*-ID database and the low similarity percentages may indicate that there exists a variety of closely related FFSC members within the grassland biome of South Africa. A study by Niehaus et al. (2016) found that the genes involved in producing secondary metabolites by members of the FFSC indicated to species-specific differences, and can be related to host specificity. The gene region, such as the *tef1-*α gene region, has shown some difficulties in resolving recently evolved sister species in the FFSC for instance, and the inclusion of additional gene regions will help to resolve these phylogenetic groupings (O’Donnell et al., 2012, 2015).

The FCSC isolates from the current study were predominantly isolated from site 1’s large soil particles, with only one FCSC isolate obtained from site 2’s small soil particles. PPRI 26282, was the only isolate to produce an identical MLST against both databases and identified as FCSC MLST 1-m. The nBLAST™ result for the remaining FCSC isolates were identical to each other but differed between the respective databases, FCSC MLST 5-a and MLST 2-a. The differences in identifications based on the nBLAST™ results were supported by the phylogenetic clustering with the PPRI 26282 isolate being closely related to the remaining FCSC PPRI isolates supporting their distinction as FCSC. The low resolving capabilities of the minor species complex tree was evident in the FCSC. Two reference isolates circumscribed as FCSC, *F*. *nelsonii* (NRRL 13338) and *Fusarium* sp. (NRRL 46670) (Laurence et al., 2016), were included in this study. The minor species complex tree showed that these two reference isolates formed a sister clade with the novel *F. aywerte* Species Complex (FASC), as expected from the article by Laurence et al. (2016). The minor species complex tree resolved the FCSC reference isolates in a single, well supported clade, and included the FCSC PPRI isolates. This indicates the *tef1-*α gene region as being more effective at resolving taxa belonging to the same species complex and the minor species complex tree may have just included too many species complexes decreasing its resolution capabilities among closely related complexes (O’Donnell et al., 2013, 2015). The use of the MLST typing scheme is highly effective for clinical identification in both the FIESC and FCSC, and identifications within these complexes can be done by implementing partial *tef1-*α gene regions (O’Donnell et al., 2009).

The FSSC MLST types identified from the current study have been reported as human pathogens originating from the USA (Texas, Florida and South Dakota) with several being resistant to antifungal treatments (O’Donnell et al., 2008; Rosa et al., 2019). Published FSSC studies predominantly focus on clinical and agricultural isolates, with little information on the population structure in natural ecosystems (O’Donnell et al., 2008). The recent study by Mavhunga (2021) characterised 62 FSSC isolates from soils of South African nature reserves. The analysis separated them into the three known/proposed FSSC clades. Three isolates, MLST 5-c, 5-d and 5-i, from Mavhunga (2021) grouped within clade 3 and were overall identified as three species, two of which were the first to be reported in South Africa. The same MLST identifications were made in this study from the GGHNP and based on their phylogenetic grouping within our analysis, it may be likely that they would also have resolved within clade 3, though further phylogenetic analyses would be required to confirm this. Laurence et al. (2016) included *F*. *solani* reference isolates in the *RPB1* and *RPB2* analysis that showed the FSSC grouping basal to the outgroup, similarly to the current study.

The FNSC in the minor species complex analysis comprised two PPRI isolates from the current study with identifications linked to *F*. *lyarnte* and *F*. *gaditjirri*. Both *F*. *lyarnte* (Walsh et al., 2010) and *F*. *gaditjirri* (Phan et al., 2004) were isolated from tropical grasses in Australia, and belong to the *F*. *nisikadoi* species complex (FNSC) (Laurence et al., 2016; Geiser et al., 2020). The FNSC was originally referred to as the *F*. *nisikadoi*-*F*. *miscanthi* clade (Gams et al., 1999), which comprises these two closely related species that were host-associated with Asian grasses and are able to produce pyriform conidia (Gams et al., 1999; Baayen et al., 2001). Summerell et al. (2011) suggested that both *F*. *lyarnte* and *F*. *gaditjirri* may be endophytic, with no direct pathogenic effects detected in the host plant.

## Conclusion

The current study aimed to expand on the known *Fusarium* species diversity found in South African grassland soils with low anthropogenic activity, such as in the GGHNP. Information from the current study will further contribute to the link between known pathogenic species complexes from highly cultivated soils in comparison to native soils, assisting with conservation strategies to prevent future outbreaks or for establishing new areas for use in agriculture.

## Data availability statement

The datasets presented in this study can be found in online repositories. The names of the repository/repositories and accession number(s) can be found below: https://www.ncbi.nlm.nih.gov/genbank/, OL782317–OL782573.

## Author contributions

CS wrote the manuscript. AJ, EV, and BS performed the review editing, developed the experimental design and project management, and critically reviewed the manuscript. CS and AJ contributed to the data explanation and the formal analysis. AJ, EV, and BS developed the experimental design and project management and critically reviewed the manuscript. All authors read and agreed to the published version of the manuscript.

## Footnotes

1
https://www.fusarium.org/

2
http://fusarium.mycobank.org

3
https://www.phylo.org/

4
http://tree.bio.ed.ac.uk/software/figtree/
