# Supplementary material for: Fusarium diversity from the Golden Gate Highlands National Park
Source: Front Microbiol. 2023 Apr 13;14:1149853. doi: 10.3389/fmicb.2023.1149853 (PMC10133521; doi:10.3389/fmicb.2023.1149853)
Supplement: Supplementary file 1 [file Data_Sheet_1.pdf]

Genbank Proof of Deposits

BankIt2527945 PPRI_27425	OL782317
BankIt2527945 PPRI_26266	OL782318
BankIt2527945 PPRI_27426	OL782319
BankIt2527945 PPRI_26314	OL782320
BankIt2527945 PPRI_26299	OL782321
BankIt2527945 PPRI_27406	OL782322
BankIt2527945 PPRI_27523	OL782323
BankIt2527945 PPRI_27424	OL782324
BankIt2527945 PPRI_25500	OL782325
BankIt2527945 PPRI_24788	OL782326
BankIt2527945 PPRI_25048	OL782327
BankIt2527945 PPRI_25490	OL782328
BankIt2527945 PPRI_25505	OL782329
BankIt2527945 PPRI_25483	OL782330
BankIt2527945 PPRI_25487	OL782331
BankIt2527945 PPRI_25486	OL782332
BankIt2527945 PPRI_28106	OL782333
BankIt2527945 PPRI_26771	OL782334
BankIt2527945 PPRI_26278	OL782335
BankIt2527945 PPRI_27145	OL782336
BankIt2527945 PPRI_27128	OL782337
BankIt2527945 PPRI_26246	OL782338
BankIt2527945 PPRI_27326	OL782339
BankIt2527945 PPRI_26311	OL782340
BankIt2527945 PPRI_26789	OL782341
BankIt2527945 PPRI_26275	OL782342
BankIt2527945 PPRI_26795	OL782343
BankIt2527945 PPRI_26319	OL782344
BankIt2527945 PPRI_26782	OL782345
BankIt2527945 PPRI_26243	OL782346

BankIt2527945	PPRI_26310	OL782347
BankIt2527945	PPRI_26330	OL782348
BankIt2527945	PPRI_26340	OL782349
BankIt2527945	PPRI_27322	OL782350
BankIt2527945	PPRI_26792	OL782351
BankIt2527945	PPRI_26284	OL782352
BankIt2527945	PPRI_26285	OL782353
BankIt2527945	PPRI_27530	OL782354
BankIt2527945	PPRI_26282	OL782355
BankIt2527945	PPRI_27516	OL782356
BankIt2527945	PPRI_27412	OL782357
BankIt2527945	PPRI_27417	OL782358
BankIt2527945	PPRI_27419	OL782359
BankIt2527945	PPRI_27506	OL782360
BankIt2527945	PPRI_24778	OL782361
BankIt2527945	PPRI_24781	OL782362
BankIt2527945	PPRI_26306	OL782363
BankIt2527945	PPRI_25044	OL782364
BankIt2527945	PPRI_25507	OL782365
BankIt2527945	PPRI_25502	OL782366
BankIt2527945	PPRI_25046	OL782367
BankIt2527945	PPRI_25484	OL782368
BankIt2527945	PPRI_24777	OL782369
BankIt2527945	PPRI_24786	OL782370
BankIt2527945	PPRI_26325	OL782371
BankIt2527945	PPRI_25047	OL782372
BankIt2527945	PPRI_25045	OL782373
BankIt2527945	PPRI_26270	OL782374
BankIt2527945	PPRI_25479	OL782375
BankIt2527945	PPRI_25481	OL782376
BankIt2527945	PPRI_25482	OL782377

BankIt2527945	PPRI_25496	OL782378
BankIt2527945	PPRI_26271	OL782379
BankIt2527945	PPRI_26273	OL782380
BankIt2527945	PPRI_26274	OL782381
BankIt2527945	PPRI_26332	OL782382
BankIt2527945	PPRI_24780	OL782383
BankIt2527945	PPRI_26269	OL782384
BankIt2527945	PPRI_26786	OL782385
BankIt2527945	PPRI_26238	OL782386
BankIt2527945	PPRI_27138	OL782387
BankIt2527945	PPRI_26793	OL782388
BankIt2527945	PPRI_27119	OL782389
BankIt2527945	PPRI_26268	OL782390
BankIt2527945	PPRI_24783	OL782391
BankIt2527945	PPRI_27496	OL782392
BankIt2527945	PPRI_27508	OL782393
BankIt2527945	PPRI_27528	OL782394
BankIt2527945	PPRI_27525	OL782395
BankIt2527945	PPRI_27546	OL782396
BankIt2527945	PPRI_27141	OL782397
BankIt2527945	PPRI_27414	OL782398
BankIt2527945	PPRI_27405	OL782399
BankIt2527945	PPRI_27418	OL782400
BankIt2527945	PPRI_27537	OL782401
BankIt2527945	PPRI_27511	OL782402
BankIt2527945	PPRI_27420	OL782403
BankIt2527945	PPRI_27408	OL782404
BankIt2527945	PPRI_27497	OL782405
BankIt2527945	PPRI_27519	OL782406
BankIt2527945	PPRI_27520	OL782407
BankIt2527945	PPRI_27541	OL782408

BankIt2527945	PPRI_27499	OL782409
BankIt2527945	PPRI_27403	OL782410
BankIt2527945	PPRI_27524	OL782411
BankIt2527945	PPRI_28107	OL782412
BankIt2527945	PPRI_27503	OL782413
BankIt2527945	PPRI_27404	OL782414
BankIt2527945	PPRI_27505	OL782415
BankIt2527945	PPRI_27509	OL782416
BankIt2527945	PPRI_27413	OL782417
BankIt2527945	PPRI_27513	OL782418
BankIt2527945	PPRI_27515	OL782419
BankIt2527945	PPRI_27427	OL782420
BankIt2527945	PPRI_27527	OL782421
BankIt2527945	PPRI_27533	OL782422
BankIt2527945	PPRI_27534	OL782423
BankIt2527945	PPRI_27536	OL782424
BankIt2527945	PPRI_27547	OL782425
BankIt2527945	PPRI_27130	OL782426
BankIt2527945	PPRI_27134	OL782427
BankIt2527945	PPRI_27135	OL782428
BankIt2527945	PPRI_27151	OL782429
BankIt2527945	PPRI_27429	OL782430
BankIt2527945	PPRI_27507	OL782431
BankIt2527945	PPRI_27542	OL782432
BankIt2527945	PPRI_26788	OL782433
BankIt2527945	PPRI_27162	OL782434
BankIt2527945	PPRI_26312	OL782435
BankIt2527945	PPRI_27422	OL782436
BankIt2527945	PPRI_27324	OL782437
BankIt2527945	PPRI_27543	OL782438
BankIt2527945	PPRI_27502	OL782439

BankIt2527945	PPRI_27532	OL782440
BankIt2527945	PPRI_26304	OL782441
BankIt2527945	PPRI_27544	OL782442
BankIt2527945	PPRI_27545	OL782443
BankIt2527945	PPRI_27421	OL782444
BankIt2527945	PPRI_27510	OL782445
BankIt2527945	PPRI_27514	OL782446
BankIt2527945	PPRI_25489	OL782447
BankIt2527945	PPRI_24779	OL782448
BankIt2527945	PPRI_27501	OL782449
BankIt2527945	PPRI_26276	OL782450
BankIt2527945	PPRI_26301	OL782451
BankIt2527945	PPRI_25504	OL782452
BankIt2527945	PPRI_26307	OL782453
BankIt2527945	PPRI_25497	OL782454
BankIt2527945	PPRI_24784	OL782455
BankIt2527945	PPRI_25488	OL782456
BankIt2527945	PPRI_25491	OL782457
BankIt2527945	PPRI_26281	OL782458
BankIt2527945	PPRI_26303	OL782459
BankIt2527945	PPRI_27531	OL782460
BankIt2527945	PPRI_27498	OL782461
BankIt2527945	PPRI_27518	OL782462
BankIt2527945	PPRI_27538	OL782463
BankIt2527945	PPRI_27409	OL782464
BankIt2527945	PPRI_27504	OL782465
BankIt2527945	PPRI_27512	OL782466
BankIt2527945	PPRI_27521	OL782467
BankIt2527945	PPRI_27500	OL782468
BankIt2527945	PPRI_27428	OL782469
BankIt2527945	PPRI_25501	OL782470

BankIt2527945	PPRI_25499	OL782471
BankIt2527945	PPRI_26242	OL782472
BankIt2527945	PPRI_27146	OL782473
BankIt2527945	PPRI_26331	OL782474
BankIt2527945	PPRI_26785	OL782475
BankIt2527945	PPRI_27325	OL782476
BankIt2527945	PPRI_27430	OL782477
BankIt2527945	PPRI_27529	OL782478
BankIt2527945	PPRI_27535	OL782479
BankIt2527945	PPRI_27539	OL782480
BankIt2527945	PPRI_27110	OL782481
BankIt2527945	PPRI_27127	OL782482
BankIt2527945	PPRI_26315	OL782483
BankIt2527945	PPRI_27136	OL782484
BankIt2527945	PPRI_27142	OL782485
BankIt2527945	PPRI_26320	OL782486
BankIt2527945	PPRI_27328	OL782487
BankIt2527945	PPRI_26249	OL782488
BankIt2527945	PPRI_27150	OL782489
BankIt2527945	PPRI_26308	OL782490
BankIt2527945	PPRI_27159	OL782491
BankIt2527945	PPRI_26289	OL782492
BankIt2527945	PPRI_26783	OL782493
BankIt2527945	PPRI_26781	OL782494
BankIt2527945	PPRI_26335	OL782495
BankIt2527945	PPRI_25494	OL782496
BankIt2527945	PPRI_27144	OL782497
BankIt2527945	PPRI_26336	OL782498
BankIt2527945	PPRI_26318	OL782499
BankIt2527945	PPRI_26309	OL782500
BankIt2527945	PPRI_26333	OL782501

BankIt2527945	PPRI_25480	OL782502
BankIt2527945	PPRI_25493	OL782503
BankIt2527945	PPRI_26267	OL782504
BankIt2527945	PPRI_26323	OL782505
BankIt2527945	PPRI_26313	OL782506
BankIt2527945	PPRI_26283	OL782507
BankIt2527945	PPRI_26326	OL782508
BankIt2527945	PPRI_26334	OL782509
BankIt2527945	PPRI_26317	OL782510
BankIt2527945	PPRI_26322	OL782511
BankIt2527945	PPRI_27125	OL782512
BankIt2527945	PPRI_26280	OL782513
BankIt2527945	PPRI_27149	OL782514
BankIt2527945	PPRI_26339	OL782515
BankIt2527945	PPRI_26251	OL782516
BankIt2527945	PPRI_25049	OL782517
BankIt2527945	PPRI_26328	OL782518
BankIt2527945	PPRI_27124	OL782519
BankIt2527945	PPRI_27118	OL782520
BankIt2527945	PPRI_26279	OL782521
BankIt2527945	PPRI_26286	OL782522
BankIt2527945	PPRI_26288	OL782523
BankIt2527945	PPRI_26338	OL782524
BankIt2527945	PPRI_24787	OL782525
BankIt2527945	PPRI_24785	OL782526
BankIt2527945	PPRI_26327	OL782527
BankIt2527945	PPRI_26799	OL782528
BankIt2527945	PPRI_26337	OL782529
BankIt2527945	PPRI_26784	OL782530
BankIt2527945	PPRI_26245	OL782531
BankIt2527945	PPRI_27137	OL782532

BankIt2527945	PPRI_26247	OL782533
BankIt2527945	PPRI_26248	OL782534
BankIt2527945	PPRI_27160	OL782535
BankIt2527945	PPRI_26290	OL782536
BankIt2527945	PPRI_26291	OL782537
BankIt2527945	PPRI_27331	OL782538
BankIt2527945	PPRI_26324	OL782539
BankIt2527945	PPRI_26265	OL782540
BankIt2527945	PPRI_26341	OL782541
BankIt2527945	PPRI_26264	OL782542
BankIt2527945	PPRI_26287	OL782543
BankIt2527945	PPRI_27153	OL782544
BankIt2527945	PPRI_26240	OL782545
BankIt2527945	PPRI_26241	OL782546
BankIt2527945	PPRI_26272	OL782547
BankIt2527945	PPRI_26796	OL782548
BankIt2527945	PPRI_26277	OL782549
BankIt2527945	PPRI_26772	OL782550
BankIt2527945	PPRI_27329	OL782551
BankIt2527945	PPRI_26302	OL782552
BankIt2527945	PPRI_25495	OL782553
BankIt2527945	PPRI_26316	OL782554
BankIt2527945	PPRI_27132	OL782555
BankIt2527945	PPRI_27431	OL782556
BankIt2527945	PPRI_24782	OL782557
BankIt2527945	PPRI_25485	OL782558
BankIt2527945	PPRI_26250	OL782559
BankIt2527945	PPRI_26263	OL782560
BankIt2527945	PPRI_26305	OL782561
BankIt2527945	PPRI_26239	OL782562
BankIt2527945	PPRI_26329	OL782563

BankIt2527945	PPRI_27526	OL782564
BankIt2527945	PPRI_27540	OL782565
BankIt2527945	PPRI_26321	OL782566
BankIt2527945	PPRI_26300	OL782567
BankIt2527945	PPRI_27410	OL782568
BankIt2527945	PPRI_27327	OL782569
BankIt2527945	PPRI_27432	OL782570
BankIt2527945	PPRI_25492	OL782571
BankIt2527945	PPRI_25509	OL782572
BankIt2527945	PPRI_25506	OL782573
