# Supplementary figures and images for: Fusarium diversity from the Golden Gate Highlands National Park
Source: Front Microbiol. 2023 Apr 13;14:1149853. doi: 10.3389/fmicb.2023.1149853 (PMC10133521; doi:10.3389/fmicb.2023.1149853)

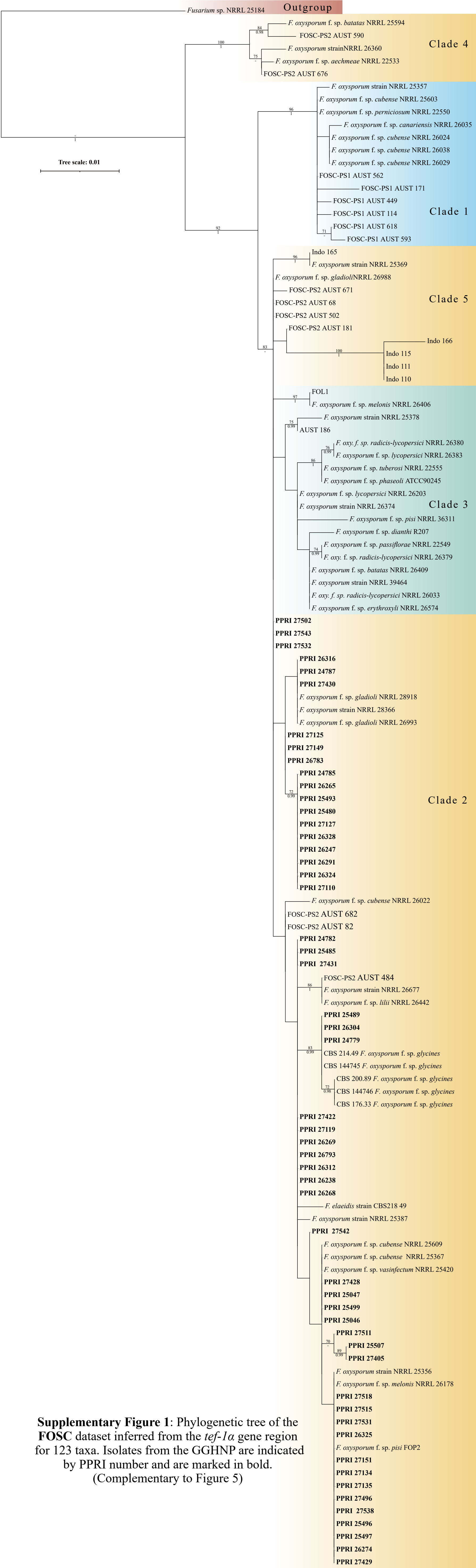

Supplement: Supplementary file 2 [file Image_1.pdf]

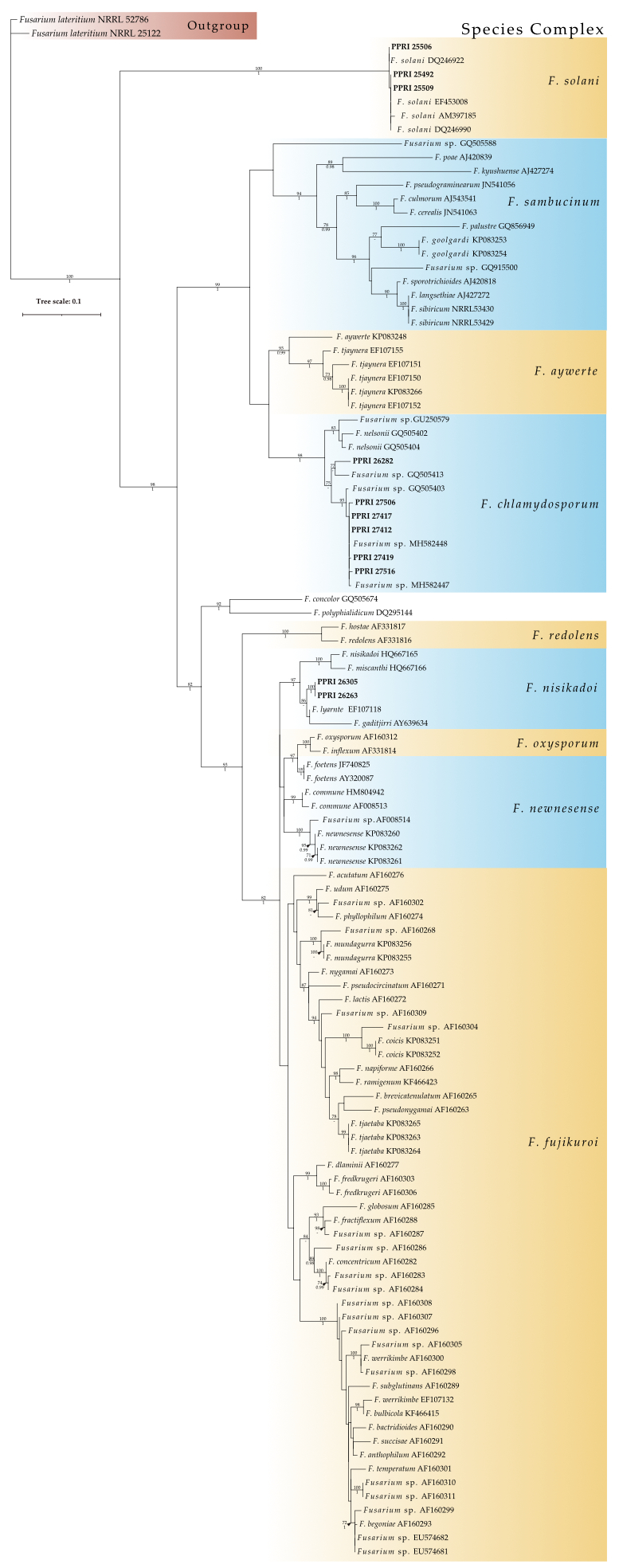

Supplement: Supplementary file 6 [file Image_5.JPEG]
