# Supplementary material for: Fusarium diversity from the Golden Gate Highlands National Park
Source: Front Microbiol. 2023 Apr 13;14:1149853. doi: 10.3389/fmicb.2023.1149853 (PMC10133521; doi:10.3389/fmicb.2023.1149853)
Supplement: Supplementary file 3 [file Image_2.pdf]

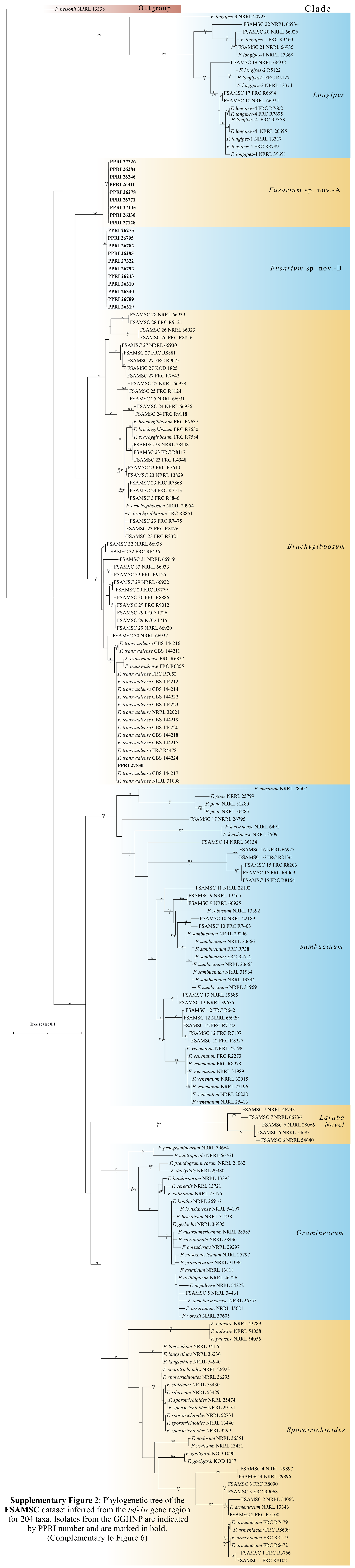

Supplementary Figure 2: Phylogenetic tree of the **FSAMSC** dataset inferred from the *tef-1α* gene region for 204 taxa. Isolates from the GGHNP are indicated by PPRI number and are marked in bold. (Complementary to Figure 6)
