# Supplementary material for: Fusarium diversity from the Golden Gate Highlands National Park
Source: Front Microbiol. 2023 Apr 13;14:1149853. doi: 10.3389/fmicb.2023.1149853 (PMC10133521; doi:10.3389/fmicb.2023.1149853)
Supplement: Supplementary file 5 [file Image_4.pdf]

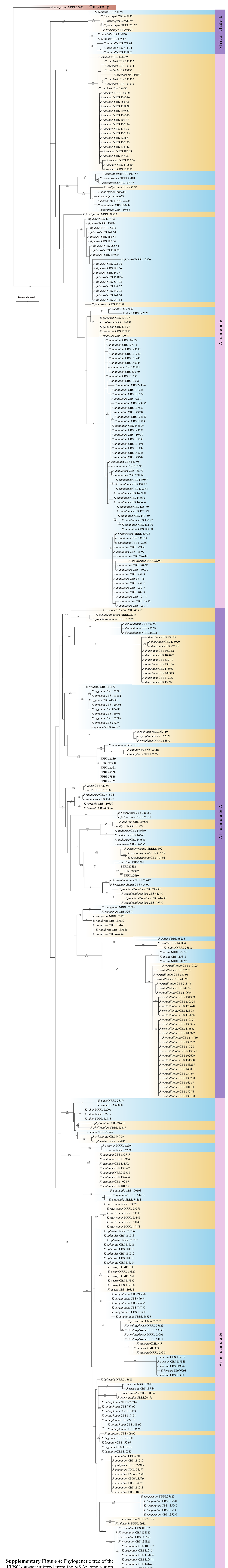

Supplementary Figure 4: Phylogenetic tree of the **FFSC** dataset inferred from the *tef-1α* gene region for 359 taxa. Isolates from the GGHNP are indicated by PPRI number and are marked in bold. (Complementary to Figure 8)
